# Clinical Outcomes of Multidisciplinary Remote Patient Monitoring for Patients With Poorly Controlled Type 2 Diabetes: Retrospective Cohort Study

**DOI:** 10.2196/105115

**Published:** 2026-11-09

**Authors:** Carly Brown, Sachin Karki, Vishnu Garla

**Affiliations:** 1 IMPACT Center for Pharmacy Transformation, School of Pharmacy, University of Mississippi, 2500 North State Street, Jackson, MS, 39216, United States, 1 6019844175; 2 Center for Telehealth and Emerging Technologies, University of Mississippi Medical Center, Jackson, MS, United States; 3 Department of Medicine, University of Mississippi Medical Center, Jackson, MS, United States

**Keywords:** type 2 diabetes mellitus, remote patient monitoring, telemedicine, pharmacists, multidisciplinary care

## Abstract

**Background:**

Multidisciplinary remote patient monitoring (RPM) programs may support diabetes management by enabling ongoing glucose monitoring and timely treatment adjustments between clinical encounters. Evidence describing real-world implementation of RPM programs for diabetes in high-risk populations remains limited.

**Objective:**

This study evaluated clinical outcomes of a multidisciplinary RPM program for patients with uncontrolled type 2 diabetes mellitus (T2DM).

**Methods:**

Adults with T2DM, hemoglobin A_1c_ (HbA_1c_) >7%, and insulin therapy who enrolled in an RPM program at the University of Mississippi Medical Center between July 2020 and December 2022 were included. Patients received a Bluetooth-enabled glucometer and structured follow-up from nurses and pharmacists operating under a collaborative practice agreement. A propensity score–matched control group receiving usual outpatient care was identified. The primary outcome was the change in HbA_1c_ from baseline to follow-up. Secondary outcomes included acute care use and RPM engagement, including program completion.

**Results:**

Among 109 RPM participants, mean HbA_1c_ decreased from 10.79% (SD 2.35%) to 8.85% (−1.94 percentage points). In the matched cohort of 95 RPM and 95 control patients, HbA_1c_ decreased by 1.97 versus 1.71 percentage points, respectively (between-group difference: −0.27, 95% CI −1.01 to 0.47; *P*=.48). Among matched RPM participants, program completers (n=19) had greater HbA_1c_ reductions than noncompleters (n=76; −3.41 vs −1.61 percentage points; *P*=.01). Acute care use occurred in 13.7% of RPM participants versus 24.2% of matched controls; rates were 23.4 versus 24.2 per 100 person-years, respectively.

**Conclusions:**

Substantial improvements in glycemic control were observed among patients with uncontrolled T2DM enrolled in a multidisciplinary RPM program. Greater HbA_1c_ reductions were observed among program completers, although this exploratory finding may reflect response-related selection because program completion required achievement of the program’s glycemic target. Further study is needed to determine the comparative effectiveness of RPM and strategies to sustain patient engagement.

## Introduction

Type 2 diabetes mellitus (T2DM) is a leading cause of morbidity, mortality, and health care use in the United States, with a disproportionate burden in Mississippi. The state consistently ranks among the highest in T2DM prevalence and diabetes-related complications, including hospitalizations and cardiovascular disease, contributing to substantial health care expenditures and strain on the health care system [1,2].

These challenges are further amplified by Mississippi’s predominantly rural landscape and limited health care workforce. Access to primary and specialty care remains constrained, with one of the lowest per capita supplies of primary care physicians in the United States, particularly in rural areas [3]. Limited access to timely care may contribute to delayed treatment intensification, poor glycemic control, and increased risk of preventable complications, highlighting the limitations of traditional, clinic-based models of diabetes management for high-risk populations.

Remote patient monitoring (RPM) has emerged as a promising strategy to improve chronic disease management by enabling real-time transmission of blood glucose (BG) data, facilitating timely clinical intervention, and supporting patient engagement. Randomized controlled trials and meta-analyses have demonstrated that telehealth and RPM interventions can improve glycemic control in patients with T2DM, although the magnitude of effect varies across implementation models and patient populations [4-6]. Much of the existing evidence is derived from controlled settings or narrowly defined populations, with less evidence from real-world implementation among patients with poorly controlled T2DM and substantial barriers to accessing traditional care.

Collaborative care models incorporating pharmacists, nurses, and physicians have been shown to improve diabetes outcomes through coordinated care delivery, medication optimization, and patient education [7,8]. Pharmacist-led interventions in particular have demonstrated clinically meaningful reductions in hemoglobin A_1c_ (HbA_1c_), especially when supported by collaborative practice agreements (CPAs) that enable timely medication intensification [8]. Integrating these roles within an RPM framework may further enhance care delivery by combining ongoing remote monitoring with proactive, coordinated clinical management between traditional clinic visits.

Despite growing interest in RPM, evidence describing multidisciplinary RPM programs in medically underserved populations, particularly those incorporating pharmacist-led medication management, remains limited. In addition, comparative evidence from routine clinical practice is less well established, and less is known about how patient engagement within RPM relates to glycemic outcomes. Therefore, the objective of this study was to evaluate changes in glycemic control among patients with uncontrolled T2DM enrolled in a multidisciplinary RPM program compared with patients receiving usual care in a real-world setting. We also examined the association of RPM engagement and program completion with changes in glycemic control among the RPM cohort.

## Methods

### Study Design and Setting

This retrospective cohort study was conducted at the University of Mississippi Medical Center (UMMC) using data extracted from electronic health records. The umbrella study protocol included patients from 2018 through 2022; however, the present diabetes analysis was restricted to patients enrolled in the UMMC RPM program between July 2020 and December 2022, during which the pharmacist-integrated diabetes RPM model and standardized clinical workflows and protocols were implemented in routine clinical practice. Patients were eligible for inclusion if they had T2DM with HbA_1c_ >7% and were prescribed insulin therapy at the time of enrollment. The RPM program prioritized insulin-treated patients due to their higher risk of glycemic variability and need for frequent medication titration.

### RPM Intervention

Patients in the RPM program received a Bluetooth-enabled glucometer, test strips, and a tablet device for transmitting daily BG readings and completing survey questions on medication adherence, side effects, and nonpharmacologic behaviors such as diet and exercise. After receiving the telemonitoring kit, patients were contacted by technical support provided by the RPM vendor to assist with setup.

Each patient was assigned a registered nurse (RN) who served as the primary point of contact throughout participation. During onboarding, the RN introduced the program, provided education on recognizing and managing hyperglycemia and hypoglycemia, and developed an individualized care plan. The pharmacist then conducted an onboarding call to explain the CPA, review the goals of the program, perform medication reconciliation, and identify potential barriers to medication adherence.

After onboarding, nurses monitored incoming BG data daily through a secure dashboard and contacted patients for critical values, defined as a BG level >450 mg/dL or <70 mg/dL. Safety protocols required escalation to the pharmacist if 2 unexplained critical values occurred within 7 days. Structured BG review calls were conducted every 2 weeks, during which medication therapy and adherence were assessed, and treatment adjustments were considered.

For the primary comparative analysis, RPM exposure was defined as enrollment in the RPM program and receipt of the telemonitoring equipment, regardless of whether the patient subsequently completed a scheduled biweekly BG review.

Engagement with the RPM program was evaluated separately as a secondary outcome. Patients who completed at least 1 scheduled biweekly BG review with the clinical team were considered to have engaged with the clinical component of the program, as these reviews represented the primary opportunity for medication adjustment and clinical intervention within the RPM workflow. Nurses followed up with patients meeting glycemic goals or requiring reengagement due to insufficient monitoring, while the pharmacist contacted patients who were not meeting goals. Patients were expected to check preprandial BG levels 3 to 4 times per day and to have at least 8 days of measurements within each 2-week review period to meet monitoring requirements and be eligible for medication titration.

The program’s glycemic target was a mean preprandial BG level of <140 mg/dL. For patients not meeting goals, pharmacists implemented therapy adjustments using a structured medication intensification algorithm adapted from the American Diabetes Association Standards of Care [9]. All medication changes were made under the CPA, and both the referring physician or nurse practitioner and collaborating endocrinologist were notified.

Patients were discharged from the RPM program upon program completion, lack of engagement, or at the patient’s request. Program completion was defined as achievement of the glycemic target (mean preprandial BG <140 mg/dL) sustained for at least 3 months.

### Control Group

The comparison group was identified from the UMMC electronic health record and consisted of adult patients with T2DM and HbA_1c_ >7% who were receiving outpatient care at UMMC during the broader study period but were not enrolled in the RPM program. The initial control source pool included 765 patients with qualifying index dates between January 1, 2018, and December 30, 2021. After application of the follow-up HbA_1c_ requirement and exclusion criteria, 729 patients remained eligible for propensity score matching. Control patients were required to have a follow-up HbA_1c_ measurement after the index date and within 1 year and were excluded if they had previously participated in an RPM program. Control patients did not receive the RPM intervention or RPM-provided telemonitoring equipment through the study program. Insulin use was not available as a reliably captured eligibility criterion for the control cohort.

Because this was a retrospective study, the specific specialty providing diabetes care, frequency and content of outpatient visits, access to glucose monitoring supplies, frequency of home glucose monitoring, and diabetes-related services received outside UMMC were not systematically captured and could not be reliably characterized. Participation in other telehealth or specialty diabetes programs outside UMMC also could not be reliably determined. The control group therefore represents usual outpatient care within the UMMC health system rather than a standardized treatment protocol.

### Outcomes

The primary outcome was the change in HbA_1c_ from baseline to follow-up. For both RPM and control patients, baseline HbA_1c_ was defined as the most recent measurement before the RPM enrollment date or control index date, respectively, provided it occurred within the preceding year. For RPM patients, follow-up HbA_1c_ was defined as the most recent measurement obtained after discharge from the RPM program and within 1 year. For control patients, follow-up HbA_1c_ was defined as the most recent measurement obtained after the index date and within 1 year. The same 1-year HbA_1c_ ascertainment windows were applied to both groups. Because laboratory testing occurred as part of routine clinical care, the actual interval between baseline and follow-up HbA_1c_ measurements varied between patients.

Secondary outcomes included all-cause acute care use and RPM engagement, including program completion. Exploratory analyses examined medication adjustments and the association between RPM engagement and HbA_1c_ change. Acute care use was defined as any emergency department visit or inpatient hospitalization recorded in the UMMC electronic health record during the applicable observation period. For control patients, acute care use was assessed during the 1-year period following the control index date. For RPM patients, acute care use was assessed during the period of RPM enrollment. Acute care use was compared between groups based on the proportion of patients experiencing at least 1 event and by calculating event rates per 100 person-years to account for differences in observation time.

Because the timing of HbA_1c_ measurements varied between patients, the baseline-to-follow-up HbA_1c_ measurement interval was evaluated as a potential source of variation in the observed change in HbA_1c_.

### Statistical Analysis

Descriptive statistics were used to summarize baseline demographic and clinical characteristics. Within-group changes in HbA_1c_ from baseline to follow-up were assessed using paired 2-tailed *t* tests, and between-group comparisons were made with a Welch 2-sample *t* test.

Propensity score matching was used to create a comparable usual care control group for the primary comparative analysis. Propensity scores were estimated using logistic regression incorporating age, sex, BMI, baseline HbA_1c_, and serum creatinine. Patients were matched 1:1 using nearest-neighbor matching within a caliper of 0.25 logit units. Covariate balance between matched groups was assessed using standardized mean differences (SMDs). The primary comparative analysis included eligible RPM enrollees regardless of subsequent RPM engagement or program completion. Patients without complete data for the variables required for propensity score matching were excluded from the matching procedure.

Because the baseline-to-follow-up HbA_1c_ measurement interval differed between groups, post hoc sensitivity analyses were conducted using linear regression to evaluate whether adjustment for this interval altered the estimated association between RPM participation and HbA_1c_ change. Three models were evaluated: (1) an unadjusted model comparing RPM and control groups, (2) a model adjusted for the time between baseline and follow-up HbA_1c_ measurements, and (3) a fully adjusted model incorporating both the time between baseline and follow-up HbA_1c_ measurements and baseline HbA_1c_. These analyses were conducted as post hoc sensitivity analyses based on differences in the timing of follow-up HbA_1c_ measurements identified during evaluation of the data.

Because the study used retrospective clinical data, some variables were incompletely documented in the electronic health record. Analyses were conducted using available data, and no imputation was performed. Due to the variability in documentation practices, medication adjustment data were only available for a subset of participants. The number of observations included in individual analyses therefore varied according to data availability.

Engagement and program completion were evaluated separately from the primary comparative analysis. Among RPM participants, HbA_1c_ changes were examined according to engagement with the RPM clinical team and program completion. These analyses were considered exploratory because engagement and program completion may be influenced by patient characteristics and other factors associated with treatment response.

Acute care use was evaluated by comparing the proportion of patients experiencing at least 1 emergency department visit or hospitalization between groups and by calculating the rates per 100 person-years to account for differences in observation time.

Statistical analyses were conducted using R (version 4.5.3; R Foundation for Statistical Computing) in RStudio (version 2026.01.1+403; Posit PBC). Propensity score matching was performed using the *MatchIt* package. Statistical significance was defined as a 2-sided *P*<.05.

### Ethical Considerations

This study was approved by the Institutional Review Board at the University of Mississippi Medical Center (UMMC-IRB-2022-399). The requirement for informed consent was waived by the institutional review board due to the retrospective nature of the study. Study data were handled in accordance with institutional requirements for protecting participant privacy and confidentiality. No participant compensation was provided.

## Results

### Participant Characteristics

A total of 109 patients with uncontrolled T2DM were enrolled in the RPM program between July 2020 and December 2022 for a mean program duration of 7.3 months. Baseline characteristics of the treatment cohort are shown in Table 1.

Patients had a mean age of 58.09 (SD 11.49) years, 76.1% (n=83) were female, and 87.2% (n=95) identified as Black or African American.

**Table 1.** Baseline characteristics of remote patient monitoring participants (N=109).

Characteristic	Participants
Age (years), mean (SD)	58.09 (11.49)
Sex, n (%)	
Female	83 (76.1)
Male	26 (23.9)
Race, n (%)	
Black or African American	95 (87.2)
White or Caucasian	14 (12.8)
BMI (kg/m^2^), mean (SD)	37.70 (10.75)
Baseline hemoglobin A_1c_ (%), mean (SD)	10.79 (2.35)
Creatinine (mg/dL), mean (SD)^a^	1.07 (0.57)

a Creatinine data were unavailable for 4 participants; the mean (SD) was calculated using 105 participants.

### Clinical Outcomes

Among all 109 RPM enrollees, mean HbA_1c_ decreased from 10.79% at baseline to 8.85% at follow-up, representing a mean reduction of 1.94 percentage points. Among RPM participants with available medication management data (n=73), patients received a mean of 3.4 medication adjustments during program participation.

### Propensity Score–Matched Analysis

Of the 109 enrolled RPM patients, 105 (96.3%) had complete data for all variables required for propensity score matching. Ninety-five RPM patients were successfully matched to 95 control patients. Before matching, the RPM and control groups differed in several baseline characteristics, most notably in baseline HbA_1c_ (mean 10.83% [SD 2.38%] vs mean 8.79% [SD 1.63%], respectively; SMD=0.998). After matching, baseline characteristics were well balanced between groups, with absolute SMDs<0.15 for all matching variables as shown in Table 2.

In the matched cohort, mean HbA_1c_ decreased from 10.72% (SD 2.29%) at baseline to 8.74% (SD 2.17%) at follow-up among RPM participants, representing a mean reduction of 1.97 (SD 2.70) percentage points. Among control patients, mean HbA_1c_ decreased from 10.52% (SD 2.21%) to 8.82% (SD 2.10%), representing a mean reduction of 1.71 (SD 2.48) percentage points. The between-group difference in HbA_1c_ change was −0.27 percentage points (95% CI −1.01 to 0.47; *P*=.48).

**Table 2.** Baseline characteristics after propensity score matching.

Characteristic	RPM^a^ group (n=95)	Control group (n=95)	SMD^b^ before matching^c^	SMD after matching
Age (years), mean (SD)	58.05 (11.59)	58.67 (11.59)	0.168	0.054
Sex, n (%)				
Female	70 (73.7)	75 (78.9)	—^d^	—
Male	25 (26.3)	20 (21.1)	0.262	0.124
BMI (kg/m^2^), mean (SD)	37.31 (10.39)	36.36 (8.49)	0.271	0.100
Baseline hemoglobin A_1c_ (%), mean (SD)	10.72 (2.29)	10.52 (2.21)	0.998	0.086
Creatinine (mg/dL), mean (SD)	0.97 (0.34)	0.98 (0.34)	0.387	0.048

a RPM: remote patient monitoring.

b SMD: standardized mean difference.

c SMDs before matching were calculated using the full eligible RPM and control cohorts (n=105 and n=729, respectively).

d Female was used as the reference category for the calculation of the SMD for sex; therefore, a separate SMD was not reported for this category.

The baseline-to-follow-up HbA_1c_ measurement interval differed between groups. Among patients with available follow-up timing data, the mean interval was 340.2 (SD 194.5) days for RPM participants and 514.4 (SD 124.4) days for controls, corresponding to 11.2 and 16.9 months, respectively (*P*<.001).

Sensitivity analyses were conducted using linear regression to account for differences in the baseline-to-follow-up HbA_1c_ measurement interval. In the unadjusted model (n=190), the estimated between-group difference in HbA_1c_ change was −0.27 percentage points (95% CI −1.01 to 0.47; *P*=.48). After adjustment for the baseline-to-follow-up HbA_1c_ measurement interval (n=167), the estimated difference was −0.64 percentage points (95% CI −1.54 to 0.26; *P*=.16). In the fully adjusted model incorporating both the follow-up interval and baseline HbA_1c_ (n=167), the estimated difference was −0.55 percentage points (95% CI −1.27 to 0.17; *P*=.13).

### RPM Engagement and Program Completion

Of the 109 RPM enrollees, 58 (53.2%) completed at least 1 scheduled biweekly BG review with the clinical team, while 51 (46.8%) were discharged before completing their first scheduled review. The 51 patients discharged before the first review had a mean HbA_1c_ reduction of 1.25 (SD 2.17) percentage points (95% CI −1.86 to −0.63; *P*<.001) and were discharged for reasons that included early program withdrawal, lack of engagement, or inability to complete device onboarding.

Among the 95 RPM participants included in the matched cohort, 19 completed the full RPM program, while 76 did not. Among completers, mean HbA_1c_ decreased by 3.41 (SD 2.62) percentage points (95% CI −4.67 to −2.15; *P*<.001), compared with a 1.61-percentage-point reduction among noncompleters (difference: −1.80 percentage points, 95% CI −3.17 to −0.42; *P*=.01).

### Acute Care Use

During the applicable observation periods, 24.2% (23/95) of control patients and 13.7% (13/95) of RPM patients experienced at least 1 emergency department visit or hospitalization. The crude odds ratio for acute care use among RPM versus control patients was 0.50 (95% CI 0.22-1.11; *P*=.10). When accounting for differences in observation time, acute care use rates were 24.2 and 23.4 events per 100 person-years in the control and RPM groups, respectively.

## Discussion

### Principal Findings

This study evaluated the clinical outcomes of an integrated RPM model for patients with poorly controlled T2DM in a real-world clinical setting. Among 109 patients enrolled in the program, mean HbA_1c_ decreased by 1.94 percentage points despite a mean baseline HbA_1c_ exceeding 10%. This substantial within-group improvement is consistent with a potential role for multidisciplinary RPM in supporting ongoing diabetes management between traditional clinical encounters among patients with marked hyperglycemia.

In the propensity score–matched analysis that included RPM enrollees regardless of subsequent engagement or program completion, HbA_1c_ decreased by 1.97 percentage points in the RPM group compared with 1.71 percentage points among matched usual care controls. Although the numerical difference favored RPM, the between-group difference was not statistically significant. These findings therefore do not establish that RPM produced greater glycemic improvement than usual care. Rather, substantial improvements in glycemic control were observed in both groups, highlighting the importance of using an appropriate comparison group when interpreting uncontrolled pre-post changes.

The baseline-to-follow-up HbA_1c_ measurement interval was shorter among RPM participants, averaging approximately 11 months compared with 17 months among matched controls. Sensitivity analyses adjusting for the measurement interval and baseline HbA_1c_ continued to favor RPM, although the between-group difference remained statistically nonsignificant. Because the same 1-year HbA_1c_ ascertainment windows were applied to both groups, the observed difference in measurement intervals likely reflects the timing of routine laboratory testing rather than differences in the prespecified window. These findings do not establish that RPM caused more timely follow-up testing, but they highlight differences in the timing of glycemic assessment between the RPM and usual care groups that may be relevant when evaluating real-world RPM outcomes.

Sustained engagement was also associated with greater HbA_1c_ improvement. Among matched RPM patients, those who completed the full program experienced a mean HbA_1c_ reduction of 3.41 percentage points compared with 1.61 percentage points among noncompleters. This finding should be interpreted cautiously because program completion was not randomly assigned, and patients who remained engaged may have differed from noncompleters in motivation, clinical stability, access to care, or other factors not captured in the retrospective analysis. Furthermore, program completion required achievement of the program’s glycemic target, creating potential response-related selection in this comparison. Nonetheless, the findings highlight sustained patient engagement as an important consideration in the implementation and evaluation of RPM programs. Nearly half of enrolled patients did not complete the first scheduled structured review, emphasizing the need for strategies to improve onboarding and retention.

Acute care use findings were exploratory. Although a smaller proportion of RPM participants experienced at least 1 emergency department visit or hospitalization compared with matched controls, the crude difference was not statistically significant. Furthermore, after accounting for differences in observation time, acute care use rates were similar between groups. These findings do not suggest a clear difference in acute care use associated with RPM, and the study was not designed or powered to evaluate health care use outcomes. Future studies with standardized follow-up periods will be needed to more definitively evaluate the impact of RPM on health care use.

The study population also represents an important real-world clinical context. Participants had markedly elevated baseline HbA_1c_ levels, and most were Black or African American. The program was implemented within a health system serving a region with substantial health care access challenges, providing real-world evidence that multidisciplinary diabetes management through RPM can be implemented among patients with considerable disease burden and potential barriers to traditional care. However, the study was not designed to evaluate health disparities, and these findings should not be interpreted as evidence that RPM reduces disparities in diabetes outcomes.

### Comparison With Prior Work

These findings build upon prior work from our institution, which used RPM and telemonitoring before the integration of a pharmacist into the care team [10]. The earlier study reported implementation feasibility and improvements in glycemic control but lacked real-time medication management through a CPA. The current collaborative care model integrating pharmacists, nurses, and physicians represents an evolution of that framework by enabling direct medication adjustments, structured follow-up, and enhanced patient support. The present study further contributes to this prior experience by incorporating a propensity score–matched usual care comparison and examining the relationship between sustained program engagement and glycemic outcomes.

The findings are also consistent with prior studies demonstrating that telehealth interventions can improve glycemic control among patients with T2DM [11]. Pharmacist-led care models have been shown to produce clinically meaningful HbA_1c_ reductions through medication optimization and adherence support [12,13]. The substantial HbA_1c_ reductions observed in both RPM and usual care patients, however, reinforce the importance of interpreting uncontrolled pre-post improvements cautiously. Regression to the mean, changes in usual care, and other clinical factors may contribute to observed improvements independent of the RPM intervention. The matched analysis provides a more informative comparison and did not demonstrate a statistically significant difference between RPM and usual care.

The multidisciplinary structure of the intervention may provide a practical mechanism for delivering more ongoing diabetes management between clinic visits. Pharmacist involvement enabled medication reconciliation, identification of medication-related barriers, and medication adjustment under a CPA, while nurses provided glucose monitoring, patient outreach, and escalation of concerning values. However, this study was not designed to determine which individual components of the multidisciplinary model contributed most to observed outcomes.

### Implications for Clinical Practice and Future Research

Rather than demonstrating superiority over usual care, this study provides real-world evidence that a multidisciplinary RPM model can be implemented in routine clinical practice and was associated with glycemic improvement among patients with poorly controlled T2DM. The shorter baseline-to-follow-up HbA_1c_ measurement interval among RPM participants also highlights the importance of accounting for real-world differences in the timing of glycemic assessment when evaluating RPM outcomes.

These findings highlight patient engagement as an important area for future implementation efforts. Strategies to improve onboarding, adherence to glucose monitoring, and retention may be particularly important given the proportion of patients who discontinued participation before receiving the full clinical intervention. Future studies should evaluate whether targeted engagement strategies improve retention and clinical outcomes and should examine comparative effectiveness, health care use, cost-effectiveness, and long-term sustainability.

### Limitations

This study has several limitations. First, the observational design limits causal inference despite the use of propensity score matching. Regression to the mean may have contributed to the observed HbA_1c_ reductions, particularly given the high baseline HbA_1c_ levels among RPM participants. Although matching balanced measured baseline characteristics, unmeasured clinical and social factors may have influenced outcomes.

Second, the usual care cohort consisted of patients receiving outpatient care within UMMC, but the available data did not allow reliable characterization of specialty care, frequency or content of outpatient visits, access to glucose monitoring supplies, home glucose monitoring practices, or diabetes-related services received outside the health system. In addition, insulin use was a criterion for RPM enrollment but was not reliably available for the control cohort, preventing confirmation that the groups had comparable insulin treatment profiles. Differences in these factors may have contributed to observed outcomes and limit attribution of between-group differences specifically to RPM.

Third, the actual interval between baseline and follow-up HbA_1c_ measurements varied between groups, and measurement dates were incompletely available for some patients. Although sensitivity analyses accounted for this measurement interval and baseline HbA_1c_, these analyses involved a smaller analytic sample and remain subject to the limitations of observational data. The relatively small final matched cohort may also have limited power to detect between-group differences.

Finally, patient engagement with RPM varied substantially. The analysis of program completion is particularly susceptible to selection bias because patients who completed the program may have differed systematically from those who discontinued participation. Because achievement of the glycemic target was part of the definition of program completion, the observed association between completion and greater HbA_1c_ reduction is also subject to response-related selection and should not be interpreted as evidence that program completion caused greater glycemic improvement. Acute care use analyses were also exploratory and limited by differences in available observation periods between groups, with control patients assessed for 1 year and RPM patients assessed during RPM enrollment.

### Conclusions

This study found substantial improvements in glycemic control among patients with poorly controlled T2DM enrolled in a multidisciplinary RPM program; however, a propensity score–matched comparison did not demonstrate a statistically significant difference in HbA_1c_ change compared with usual care. Greater HbA_1c_ reductions among program completers were associated with sustained engagement but should be interpreted cautiously because program completion required achievement of the study-defined glycemic target. Future research should evaluate strategies to improve RPM retention and determine its comparative effectiveness, impact on health care use, cost-effectiveness, and long-term sustainability.

## Abbreviations

BG: blood glucose
CPA: collaborative practice agreement
HbA_1c_: hemoglobin A_1c_
RN: registered nurse
RPM: remote patient monitoring
T2DM: type 2 diabetes mellitus
UMMC: University of Mississippi Medical Center
